# MicroRNA‐372 enhances radiosensitivity while inhibiting cell invasion and metastasis in nasopharyngeal carcinoma through activating the PBK‐dependent p53 signaling pathway

**DOI:** 10.1002/cam4.1924

**Published:** 2019-01-18

**Authors:** Zhe Wang, Ji‐Wei Mao, Guang‐Yan Liu, Fu‐Guang Wang, Zai‐Shuang Ju, Dong Zhou, Ruo‐Yu Wang

**Affiliations:** ^1^ Department of Medical Oncology Affiliated Zhongshan Hospital of Dalian University Dalian China; ^2^ The Key Laboratory of Biomarker High Throughput Screening and Target Translation of Breast and Gastrointestinal Tumor Dalian University Dalian China; ^3^ College of Basic Medical Sciences Shenyang Medical College Shenyang China

**Keywords:** invasion, metastasis, microRNA‐372, nasopharyngeal carcinoma, p53 signaling pathway, PDZ‐binding kinase, radiosensitivity

## Abstract

Nasopharyngeal carcinoma (NPC) is a common cancer found in the nasopharynx, which plagues countless NPC patients. MicroRNA‐372 (miR‐372) has been reported to be involved in various tumors. Here, we explored the important role of miR‐372 in radiosensitivity, invasion, and metastasis of NPC. Microarray analysis was conducted to search the NPC‐related differentially expressed genes (DEGs) and predict the miRs regulating PBK, which suggested that miR‐372 could influence the development of NPC via PBK and the p53 signaling pathway. Importantly, miR‐372 was observed to target PBK, thus down‐regulating its expression. Then, NPC 5‐8F and C666‐1 cells were selected, and treated with ionization radiation and alteration of miR‐372 and PBK expression to explore the functional role of miR‐372 in NPC. The expression of miR‐372, PBK, Bcl‐2, p53, and Bax as well as the extent of Akt phosphorylation were measured. In addition, cell colony formation, cell cycle, proliferation, apoptosis, migration, and invasion were detected. At last, tumor growth and the effect of miR‐372 on radiosensitivity of NPC were evaluated. Besides, over‐expressed miR‐372 down‐regulated Bcl‐2 and PBK expression and the extent of Akt phosphorylation while up‐regulated the expression of p53 and Bax. Additionally, miR‐372 over‐expression and radiotherapy inhibited cell clone formation, proliferation, tumor growth, migration, invasion, and cell cycle entry, but promoted cell apoptosis. However, the restoration of PBK in NPC cells expressing miR‐372 reversed the anti‐tumor effect of miR‐372 and activation of the p53 signaling pathway. In conclusion, the study shows that up‐regulated miR‐372 promotes radiosensitivity by activating the p53 signaling pathway via inhibition of PBK.

## 1 INTRODUCTION

Nasopharyngeal carcinoma (NPC), a highly metastasizing and invasive cancer, is widespread in southern China and southeast Asia with environmental influence, Epstein‐Barr virus infection, and genetic factors serving as the main inducements.^1^ Besides, NPC is often found in epithelial cells with a particular endemic distribution, and its mortality is greatly reduced by effective systemic agents, population screening as well as radiotherapy.^2^ Although NPC patients diagnosed at early stages may be effectively cured by radiotherapy, NPC patients at advanced stage still face unsatisfactory treatment efficacy owing to frequently occurred metastasis and local recrudesce.^3^ Radiation resistance remains to be the primary inducer of unsuccessful radiotherapy for cancer, thus increasing the tumor cell radiosensitivity is critical for improving radiotherapy efficacy and reducing the damage of normal tissue round tumor.^4^ Meanwhile, microRNAs (miRs) have been reported to affect carcinogenesis at various levels, and play an effective role in regulating tumor radiosensitivity by modulating signaling pathways associated with DNA damage repair, tumor microenvironment, apoptosis, and cell cycle checkpoint.^5^ Therefore, it is necessary to explore the effect of miRs on radiosensitivity, invasion, and metastasis of NPC and related mechanisms.

PDZ‐binding kinase (PBK) is defined as a mitogen‐activated protein kinase between MEK7 and MEK1/2, a mitosis kinase that enhances cytokinesis as well as a kinase that promotes metastasis and cell migration in lung cancer.^6^ MicroRNA‐216b (miR‐216b) was recently demonstrated to play a repressive role in tumors by regulating the expression of PBK.^7^ According to the TargetScan database, there are theoretical complementary sequences between 3′‐untranslated region (UTR) of PBK and miR‐372. MiRs have been demonstrated to participate in multiple cellular progressions such as metabolism, cell apoptosis, and proliferation.^8^ MiR‐372 functions as a tumor suppressor or a tumor promoter in numerous tumors, including gastric cancer, brain cancer, glioma, and colorectal cancer.^9^ Moreover, PBK has been proved to function with protein 53 (p53), and its over‐expression induces a poor overall survival rate of NPC, gastric cancer, and lung cancer sufferers.^10^ In addition, p53 is recognized as a transcription factor that suppresses tumor formation by reacting to DNA damage, and activating signaling pathways that induce apoptosis, inhibit cell division and repair DNA.^11^ Moreover, a recent study revealed that aberrantly expressed p53 is highly associated with tumor formation and progression of NPC.^12^ Activation of the p53 signaling pathway has been reported to promote radiosensitivity in breast cancer cell line MCF7 via over‐expressed SMAR1.^13^ Based on the above literature, a hypothesis was proposed that miR‐372 could suppress the development of NPC by regulating the p53 signaling pathway via PBK. Therefore, the current study aims to investigate the underlying molecular mechanisms of miR‐372, PBK, and the p53 signaling pathway in radiosensitivity of NPC cells, thus providing more effective therapy strategies for NPC patients.

## 2 MATERIALS AND METHODS

### 2.1 Ethical statement

The current study was carried out in strict accordance with the recommendations of the Guide for the Care and Use of Laboratory Animals of the National Institutes of Health. The protocol was approved by the Institutional Animal Care and Use Committee of our hospital, and all efforts were made to minimize the suffering of the included animals.

### 2.2 Microarray analysis

Firstly, NPC‐related chips GSE13597 and GSE12425 were retrieved and downloaded from the Gene Expression Omnibus (GEO) database (https://www.ncbi.nlm.nih.gov/geo/) with “Nasopharyngeal carcinoma” serving as the retrieval key word. GSE13597 included three normal control samples and 25 NPC samples, while GSE12425 comprised of 10 normal control samples and 31 NPC samples. Next, the limma package of R language was utilized for differential analysis with *P* value <0.05 and |logFC| > 2 as the screening threshold of DEGs. Subsequently, the pheatmap package of R language was used to plot the thermal map of the first 35 DEGs in the two chips. Venn diagrams online construction website (http://bioinformatics.psb.ugent.be/webtools/Venn/) was applied to construct Venn map and obtain the intersections of the two aforementioned chips.

DisGeNET (http://www.disgenet.org/web/DisGeNET/menu) is a discovery platform which collects various human diseases‐associated genes and variants for public use. The initial 10 obtained genes from this website with “Nasopharyngeal carcinoma” serving as the key word were included for the following experiment. STRING (https://string-db.org/) is a database which interacts the known and predicted proteins, which includes direct (physical) and indirect (functional) interaction, and protein correlation analysis on the intersection of the 10 NPC‐related genes and results from chip analysis was carried out using this database.

The miRs that potentially regulated PBK were retrieved using the miRDB (http://www.mirdb.org/) database, TargetScan (http://www.targetscan.org/vert_71/) database, microRNA.org (http://34.236.212.39/microrna/home.do) database and DIANA (http://diana.imis.athena-innovation.gr/DianaTools/index.php?r=microT_CDS/index) database by inputting PBK and selecting Human as species. Following that, a Venn diagram online construction website was applied to obtain the intersection of the predicted results from the four databases.

### 2.3 Cell culture and grouping

Two NPC cell lines, 5‐8F and C666‐1, provided by BeNa Culture Collection (BNCC) Company (Manassas, VA, USA) were cultured with Roswell Park Memorial Institute (RPMI) 1640 medium containing 10% fetal bovine serum (FBS) at 37°C with 5% CO_2_. After cell adherence, the cells were sub‐cultured, and detached using 0.25% trypsin. Then, cells at the logarithmic phase of growth were collected for the following experiment. Radiation dosage assay was used to detect the effect of radiation with various dosages on cell proliferation and clone formation ability. The cells were assigned into six groups irradiated by 0 Gy, 2 Gy, 4 Gy, 6 Gy, 8 Gy, and 10 Gy rays, respectively. The following experiment of the effect of miR‐372 and its target gene PBK on radiotherapy were conducted by adopting 4 Gy ray radiation. 5‐8F and C666‐1 cells were arranged into control group (without any treatment), blank group (treated with ionization radiation), empty vector group (treated with empty vector +ionization radiation), miR‐372 mimic group (treated with miR‐372 mimic + ionization radiation), miR‐372 inhibitor group (treated with miR‐372 inhibitor + ionization radiation), and miR‐372 mimic + PBK group (treated with miR‐372 mimic + ionization radiation + PBK). MiR‐372 mimic (sequence: GUGGGCCUCAAAUGUGGAGCACUAUUCUGAUGUCCAAGUGGAAAGUGCUGCGACAUUUGAGCGUCAC), miR‐372 inhibitor (sequence: GTGCGCTCTGTCGCGCCTTTCCCTTGGCTCGTGTGCTCCCTTTGGGCCCC) and PBK plasmid (sequence: ATGAGCGACGTGGCTATTGTGA) were purchased from Guangzhou RiboBio Co., Ltd. (Guangdong, China). Radiation was conducted at 24 hours after transfection.

### 2.4 Cell transfection

Cells were inoculated in a 50 mL culture bottle, and further cultured in complete medium until cell confluence reached 30%‐50%. Lipofectamine 2000 (Gibco Company Grand Island, NY, USA) and DNA or RNA content to be transfected were prepared in a sterile Eppendorf (EP) tube as follows: 5 µL lipofectamine 2000 was mixed with 100 µL serum‐free medium, and placed at room temperature for 5 minutes; RNA (50 nmol) or DNA (2 μg) to be transfected was mixed with 100 µL serum‐free medium, and placed at room temperature for 20 minutes to form a complex with lipidosome. The cells in the culture bottle were washed by serum‐free medium. Following that, the complex was added with serum‐free medium without penicillin/streptomycin, gently and evenly mixed, added into a 50 mL culture bottle to be transfected, and placed at 37°C in a 5% CO_2_ incubator, and then further cultured in complete medium after 6‐8 hr.

### 2.5 Dual‐luciferase reporter gene assay

TargetScan was employed in order to predict the target gene of miR‐372, and obtain the fragment sequence of action site in the gene. The full length of 3'UTR sequence (Beijing Genomics Institute, Beijing, China; binding site: AUUUGAG) of PBK was obtained by polymerase chain reaction (PCR) amplification, and cloned into the downstream of fluorescein gene in pGL3 vector (Promega Corporation, Madison, WI, USA), which was regarded as the wild type (WT)‐PBK. Quick change site‐directed mutagenesis kit (Stratagene, CedarCreek, TX, USA) was applied for mutation on the binding site between PBK and miR‐372. The mutant (MUT) 3′‐UTR (Beijing Genomics Institute, Beijing, China; binding site: GCGCACT) was cloned into pGL3 vector, which was called MT‐PBK. Then, miR‐372 and miR‐372 NC were, respectively, transfected with WT‐PBK or MT‐PBK into 5‐8F and C666‐1 cells, respectively, for 48 hours. After that, luminescence value was evaluated using a dual‐luciferase reporter system.

### 2.6 Ionization radiation

5‐8F and C666‐1 cells at the logarithmic phase of growth were collected, and placed on 1.5 cm equivalent layers when cell confluence reached 60%‐70%. The angle of the linear accelerator (Leeman Inc, Dover, DE, USA) was set to 180°. The cells were irradiated by the required dosage with 10 cm × 10 cm fields and 100 cm source‐skin distance. In addition, 1.5 cm tissue compensation gum was placed on the culture medium.

### 2.7 Clonogenic assay

5‐8F and C666‐1 cells were inoculated into two six‐well plates at a concentration of 1 × 10^6^ cells/well. One six‐well plate was cultured overnight, and irradiated at 0 Gy, 2 Gy, 4 Gy, 6 Gy, 8 Gy, and 10 Gy rays to detect the effect of radiation with various dosages on clone formation ability. The other six‐well plate was transfected according to the aforementioned method in cell grouping, and irradiated at 4 Gy ray after transfection for 24 hours. Following that, the cells were further cultured for 12 hours, fixed with cold methanol, stained with Giemsa solution for 30 minutes, naturally dried and photographed using a low power microscope to count clone number and to calculate the mean clone number. The number of formed cell colony over 50 cells was regarded as an effective bacterial colony. The experiment was repeated three times independently.

### 2.8 3‐(4,5‐dimethylthiazol‐2‐yl)‐2,5‐diphenyltetrazolium bromide (MTT) assay

5‐8F and C666‐1 cells at the logarithmic phase of growth were collected, and resuspended by cell culture medium with cell density adjusted into 1.0 × 10^4^ cells/mL. Next, the cells were uniformly seeded into a 96‐well plate (100 μL/well), and added with 10 μL MTT solution (5 mg/mL) after being irradiated with different dosages (0 Gy, 2 Gy, 4 Gy, 6 Gy, 8 Gy, and 10 Gy rays) for 48 hours. Then, the cells were cultured at 37°C for 4 hours, and dissolved with methylsulfoxide (DMSO) (Sigma‐Aldrich Chemical Company, St Louis, MO, USA). The optical density (OD) values at 490 nm were measured using a spectrophotometer, and the cell viability curve was plotted.

After that, experimentation on the effect of miR‐372 and PBK on cell proliferation was carried out. After cell adherence, the cells were transfected according to the aforementioned cell transfection method for 24 hours, irradiated at 4 Gy ray for 48 hours, added with 10 μL MTT solution (5 mg/mL), and cultured at 37°C for 4 hours, followed by dissolution with DMSO (Sigma‐Aldrich Chemical Company, St Louis, MO, USA). The OD values at 490 nm were measured using a spectrophotometer, and the cell viability curve was plotted. The experiment was repeated three times independently.

### 2.9 Western blot analysis

5‐8F and C666‐1 cells at the logarithmic phase of growth were transfected for 24 hours, and irradiated at 4 Gy ray for 48 hours, followed by removal of cell culture solution. Next, the cells were rinsed with pre‐cooling phosphate buffered saline (PBS) three times, lysed by prepared radioimmunoprecipitation assay (RIPA) lysis buffer (Beyotime Biotechnology Co., Ltd., Shanghai, China), and scraped by a cell scratcher. Then, the cell samples were placed into a 1.5 mL centrifuge tube, stirred by needles five times until fully split, centrifuged at 14 000 *g* for 10 minutes with supernatant obtained. After the concentration of the protein sample was determined by bicinchoninic acid (BCA) method, the sample was reserved at −20°C. Following that, a sodium dodecyl sulfate polyacrylamide gel electrophoresis (SDS‐PAGE) kit was used to prepare 10% separation gel and 5% spacer gel. The protein was separated using electrophoresis on polyacrylamide gel, transferred onto a nitrocellulose (NC) membrane using the wet transfer method, and sealed with 5% bovine serum albumin at room temperature for 1 hour. Subsequently, the NC membrane was added with diluted rabbit polyclonal antibody PBK (ab220106, dilution ratio of 1:250‐1:500), p53 (ab131442, dilution ratio of 1:500‐1:1000), Bcl‐2 Associated X (Bax) (ab53154, dilution ratio of 1:500‐1:1000), B‐cell lymphoma‐2 (Bcl‐2) (ab59348, dilution ratio of 1:500‐1:1000), Phosphorylated protein kinase B (p‐Akt) (ab18206, 1 μg/mL), Akt (ab8805, dilution ratio of 1:500) and Caspase‐3 (ab13847, dilution ratio of 1:500) for incubation at 4°C overnight. The above antibodies were purchased from Abcam Inc (Cambridge, MA, USA). The following day, the membrane was rinsed with phosphate buffer saline‐Tween 20 (PBST) three times (10 minutes each), incubated with secondary antibody goat‐anti‐rabbit polyclonal antibody (ab7312; Abcam) diluted with 5% skim milk, and placed on an oscillator at room temperature for 1 hours. The membrane was rinsed with PBST again three times (15 minutes each). Next, 1 mL electrochemiluminescence (ECL) reagent was prepared according to the instructions of SuperSignal^®^ West Dura Extended Duration Substrate and then added on the membrane. After treatment for 1 minutes, the excess reagent was discarded, and the membrane was sealed using a preservative film. After that, the membrane was exposed to X‐ray film for 5‐10 minutes in a dark box, followed by developing and fixation. The ratio of gray value of PBK, p53, Bax, and Bcl‐2 bands to internal reference glyceraldehyde‐3‐phosphate dehydrogenase (GAPDH) represented their respective levels. The experiment was repeated three times independently.

### 2.10 Reverse transcription quantitative polymerase chain reaction

5‐8F and C666‐1 cells at the logarithmic phase of growth were transfected for 24 hours, and irradiated at 4 Gy ray. The cells in the control group were left untreated. After that, total RNA content was extracted from the cells in each group using a miRNeasy Mini Kit (Qiagen Company, Hilden, Germany). Then, 5 μL RNA sample was collected, and diluted 20 times using RNase‐free ultrapure water. Next, OD values at excitation wavelengths of 260 and 280 nm were determined using an ultraviolet spectrophotometer, and the purity and concentration of RNA were detected. An OD260/OD280 ratio value between 1.7 and 2.1 indicated that purity was high enough for the further experimentation. Subsequently, reverse transcription was performed using a PCR amplifier to synthesize complete DNA (cDNA) template, and the primers of GAPDH, Bax, Bcl‐2, p53, PBK, U6, Akt, and miR‐372 were designed and synthesized by Shanghai Sangon Biological Engineering Technology & Services Co., Ltd. (Shanghai, China) (Table 1). Reverse transcription was carried out in accordance with the instructions of the EasyScript First‐Strand cDNA Synthesis SuperMix kit (AE301‐02; Beijing TransGen Biotech Co., Ltd., Beijing, China) employing a 20 µL system as follows: 5 μL Mix reagent was added into an EP tube, added with 5 μL total RNA and 10 μL RNase‐Free H_2_O, centrifuged, evenly mixed, and placed in the PCR amplifier (9700, Beijing Dingguo Changsheng Biotechnology Co., Ltd., Beijing, China), followed by reverse transcription. The reaction condition was as follows: 37°C for 15 minutes and 85°C for 5 seconds. Then, the samples were placed at 4°C to terminate the reaction. The generated cDNA was preserved at ‐20°C, and then collected for real‐time fluorescent quantitative PCR, which was conducted according to the instructions of the SYBR® Premix Ex TaqTM II reagent kit (RR820A, Takara Holdings Inc, Kyoto, Japan). The reaction system (total of 20 μL) included 10 μL SYBR Premix, 2 μL DNA template, 0.6 µL PCR forward primers, 0.6 µL PCR reverse primers, and 6.8 μL of sterile water. With U6 serving as the internal reference for miR‐372, and GAPDH serving as the internal reference of other genes, Reverse transcription quantitative polymerase chain reaction (RT‐qPCR) detection was carried out using ABI7500 fluorescent quantitative PCR instrument (7500, ABI Company, Oyster Bay, NY, USA) under the following reaction conditions: pre‐denaturation at 95°C for 30 seconds, 45 cycles of denaturation at 95°C for 30 seconds, annealing for 20 seconds and extension at 72°C for 30 seconds. After that, the expression of GAPDH, Bax, Bcl‐2, p53, PBK, U6, Akt, and miR‐372 was determined. Additionally, $2^{-\Delta \Delta C_{\text{t}}}$ represented the ratio of target gene expression in the experimental group to the control group, of which ΔΔ*C*_t_ = Δ*C*_t__the experimental group_ − Δ*C*_t__the control group_.^14^ *C*_t_ was the number of amplification cycle when reactive real‐time fluorescence intensity reached set threshold value. At this time, amplification was in the logarithmic phase of growth, and the experiment was repeated three times.

**Table 1:** Primer sequences for RT‐qPCR

Genes	Sequences
U6	F: 5′‐AAAGCAAATCATCGGACGACC‐3′
	R: 5′‐GTACAACACATTGTTTCCTCGGA‐3′
GAPDH	F: 5′‐TGTGGGCATCAATGGATTTGG‐3′
	R: 5′‐ACACCATGTATTCCGGGTCAAT‐3′
PBK	F: 5′‐CCAAACATTGTTGGTTATCGTGC‐3′
	R: 5′‐GGCTGGCTTTATATCGTTCTTCT‐3′
Bax	F: 5′‐CATATAACCCCGTCAACGCAG‐3′
	R: 5′‐GCAGCCGCCACAAACATAC‐3′
Bcl‐2	F: 5′‐GTCTTCGCTGCGGAGATCAT‐3′
	R: 5′‐CATTCCGATATACGCTGGGAC‐3′
p53	F: 5′‐AACTGCGGGACGAGACAGA‐3′
	R: 5′‐AGCTTCAAGAGCGACAAGTTTT‐3′
Akt	F: 5′‐CCTCCACGACATCGCACTG‐3′
	R: 5′‐TCACAAAGAGCCCTCCATTATCA‐3′
miR‐372	F: 5′‐CAACAGAAGGCTCGAGCAACCTGCGGAGAAGATAC‐3′
	R: 5′‐TTCTGATCAGGATCCCATTACAGCCAGACGCTGTAAG‐3′
Caspase3	F: 5′‐TGCCTCTTCCCCCATTCTC‐3′
	R: 5′‐GAGGTT TGCTGCATCGACAT‐3′

Akt (PKB), protein kinase B; Bax, Bcl‐2‐associated X; Bcl‐2, B‐cell lymphoma 2; F, forward; GAPDH, glyceraldehyde‐3‐phosphate dehydrogenase; p53, protein 53; PBK, PDZ‐binding kinase; R, reverse; RT‐qPCR, reverse transcription quantitative polymerase chain reaction; U6, small nuclear ribonucleic acid 6.

### 2.11 Annexin V‐fluorescein isothiocyanate/Propidium iodide staining

5‐8F and C666‐1 cells were transfected for 24 hours, irradiated at 4 Gy ray (cells in the control group remained untreated), routinely cultured for 72 hours, and collected. Next, an Annexin fluorescein isothiocyanate/Propidium iodide (FITC/PI) reagent kit (556547; Shanghai Shuojia Biotechnology Co., Ltd., Shanghai, China) was employed in order to measure cell apoptosis in all groups. Deionized water was used to dilute 10 × binding buffer into 1 × binding buffer. The cells in each group were centrifuged at 715 *g* for 5 minutes, collected, resuspended with pre‐cooled 1 × PBS, and centrifuged again at 7 *g* for 5‐10 minutes. Then, the cells were washed and suspended by adding with 300 µL 1 × binding buffer, evenly mixed with 5 µL Annexin V‐FITC, incubated at room temperature avoiding light exposure for 15 minutes, added with 5 µL PI 5 minutes before flow cytometry detection (Cube6; Partec, Munster, Germany), and ice‐bathed avoiding light exposure for 5 minutes. FITC was detected at excitation wavelengths of 480 and 530 nm, and PI was detected at an excitation wavelength over 575 nm.

### 2.12 PI staining

5‐8F and C666‐1 cells were transfected for 24 hours and irradiated at 4 Gy ray radiation (cells in the control group untreated). After routine culture for 72 hours, trypsin detachment was terminated by 10% complete medium. Next, the cells were centrifuged at 178 *g* for 5 minutes, collected, rinsed with PBS at low temperature, fixed with 75% ethanol, and incubated overnight at 4°C. Then, 1 × 10^6^ cell suspension was added with 50 mg/mL of RNase and 4 μg/mL of Hoechst33342, incubated at 37°C for 30 minutes, transferred into a flow cytometry tube, and placed in flow sorter for detection. At last, a total of 20 000 cells were counted to analyze cell proportion at various stages (G0/G1, S and G2/M) of the cell cycle. The experiment was repeated three times independently.

### 2.13 Transwell assay

5‐8F and C666‐1 cells in each group were detached by trypsin, triturated into a single cell suspension with serum‐free Dulbecco's modified eagle's medium (DMEM) and inoculated in a 24‐well plate using millicell‐inserts. After the addition of DMEM for balancing, the single cell suspension was incubated with DMEM containing FBS in the chamber at 37°C for 24 hours. The cells in the apical chamber and the extra liquid were removed using cotton buds. After that, the remaining cells were fixed with paraformaldehyde for 15 minutes, stained with crystal violet for 15 minutes and rinsed under distilled water. Lastly, the cells were observed under a microscope (BMM‐300C, Shanghai Huitong Optical Instrument Co., Ltd., Shanghai, China) to detect cell migration and count the migrating cells.

5‐8F and C666‐1 cells at the logarithmic phase of growth were transfected for 24 hours, irradiated at 4 Gy ray, and collected (cells in the control group untreated). Matrigel (Becton, Dickinson and Company, Franklin Lakes, NJ, USA) was diluted with pre‐cooling serum‐free DMEM (dilution ratio of 1:100), evenly mixed, added into all apical chambers (100 μL each) of the Transwell chamber, placed at room temperature for 2 hours, and washed with 200 μL serum‐free RPMI 1640 medium. Next, the cells were resuspended with serum‐free DMEM after cell detachment, counted, and diluted into a concentration of 3 × 10^5^ cells/mL. Then, 100 μL diluted cell suspension was added into the Transwell apical chamber (Corning Glass Works, Corning, NY, USA), while 600 μL RPMI 1640 medium containing 10% serum (chemotactic factor) was added into the basolateral chamber in accordance with the instructions of the Transwell chamber. At last, the cells were stained with crystal violet, and the number of cell across the membrane was counted. The experiment was repeated three times independently.

### 2.14 Tumor xenograft in nude mice

A total of 36 BALB/c nude mice (aged 4 weeks, weighing 17‐22 g, no limitation with sex) were purchased from Hunan SLAC Jingda Laboratory Animal Co., Ltd. (Hunan, China), and raised under specific pathogen‐free conditions, and randomly assigned into six groups (six mice per group). Next, 5‐8F cells at the logarithmic phase of growth were collected, and routinely detached by trypsin. The cell density was adjusted into 1 × 10^6^/mL. Then, 5‐8F cells were randomly divided into control group (blank control), blank group (blank control), empty vector group (transfected with empty vector), miR‐372 mimic group (transfected with miR‐372 mimic), miR‐372 inhibitor group (transfected with miR‐372 inhibitor), and miR‐372 mimic + PBK group (transfected with miR‐372 mimic + PBK). After successful transfection, the nude mice skins were disinfected, and the mice were subcutaneously inoculated with the transfected cells. The following day, the mice were subjected to radiotherapy except for those in the control group. Subsequently, tumor volume was monitored once a week and calculated using the following formula: V = π/6 (Height × length × width). The nude mice were sacrificed at the end of the 4th week, and the tumors were collected for further use.

### 2.15 Statistical analysis

All statistical analyses were performed using the SPSS 21.0 software (IBM Corp. Armonk, NY, USA). Measurement data were presented as the mean ± standard deviation. Comparisons between two groups were analyzed by* t* test, while comparisons among multiple groups were analyzed using one‐way analysis of variance (ANOVA). A value of *P < *0.05 was considered to be statistically significant.

## 3 RESULTS

### 3.1 MiR‐372 may affect the progression of NPC and regulate PBK and p53 signaling pathway

**Figure 1:**
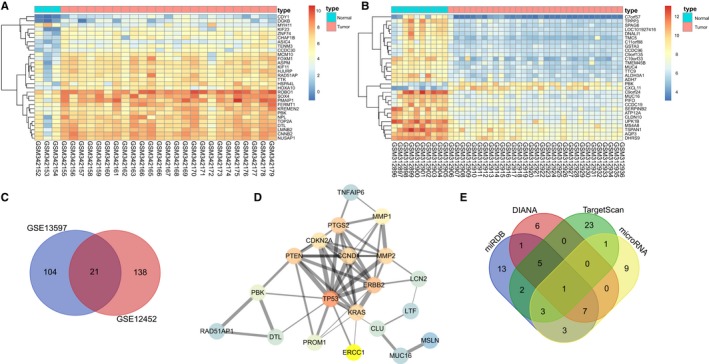
MiR‐372 is involved in NPC development and might regulate PBK. A, analysis results of NPC expression chip GSE13597; B, analysis results of NPC expression chip GSE12425; *X*‐axis represented sample number and *Y*‐axis represented genes; upper right histogram referred to the color gradation, color change from top to bottom represented expression values in descending order; each rectangle represented the level of a sample, and each column represented expression of all genes in each sample; the dendrogram in the left represented cluster analysis result of different genes from different samples; uppermost crossband represented sample type, blue referred to the normal control sample, and red referred to the tumor sample; C, Venn analysis of obtained results from GSE13597 and GSE12425 chips; blue in the left referred to the analysis result from GSE13597 chip while red in the right referred to the analysis result from GSE12452 chip, and red in the middle referred to their intersection; D, gene interactive analysis on the known NPC‐related genes and chip analysis results; circle represented gene and its color represented the correlation degree of gene, and darker color meant higher correlation degree; lines connecting two genes represented their interactive reliability, and thicker line meant higher reliability; E, predicted miRs that regulated PBK; blue represented predicted result from miRDB database, red represented predicted result from DIANA database, green represented predicted result from TargetScan database and yellow represents predicted result from microRNA.org database; the arrow in the middle represented the intersection of the predicted results from four databases; miR‐372, microRNA‐372; NPC, nasopharyngeal carcinoma; PBK, PDZ‐binding kinase

Differential analyses on NPC‐related chips GSE13597 and GSE12425 were performed using the R Programming Language. The results revealed that there were a total of 125 DEGs in GSE13597, among which 65 genes were up‐regulated while 60 genes were down‐regulated in NPC samples. Meanwhile, 159 DEGs were found in GSE12425 with 22 up‐regulated genes and 137 down‐regulated genes in NPC samples. Subsequently, the thermal maps of the first 35 DEGs in GSE13597 and GSE12425 were plotted (Figure 1A,B). The intersections of the DEGs in the two aforementioned chips were obtained through Venn map, and the results showed that there were 21 intersecting genes among the two chips (Figure 1C). In order to further select NPC‐related genes, top 10 highly correlated genes retrieved from DisGeNET database were included for the following analysis (Table 2). A total of 21 intersected genes and 10 NPC‐related genes were selected for correlation analysis (Figure 1D), and the results showed that among highly correlated genes, only PBK, MMP1, and PTGS2 were excluded from the DisGeNET database. Further analysis on these three genes revealed that MMP1 and PTGS2 have been intensively reported to be related with NPC.^15^, ^16^, ^17^, ^18^ However, the relationship between PBK and NPC remains to be rarely reported. In addition, PBK was found to be significantly up‐regulated in the NPC expression chip. Further information retrieval on the related signaling pathway of NPC revealed that the p53 signaling pathway has been demonstrated to be closely associated with the progression of NPC.^19^, ^20^, ^21^ These findings suggested that PBK might regulate NPC development through the p53 signaling pathway, but the specific molecular mechanism remained to be further confirmed. Thereby, in order to elucidate more information about the molecular mechanism of PBK in NPC, miRNA database was utilized to predict the potential miRs that regulated PBK. After that, 35 miRs were predicted in miRDB and 71 miRs in DIANA. MiRs with a score >0.85 were applied for further analyses. In addition, first 35 miRs from 269 predicted miRs in TargetScan database were analyzed, and 24 miRs were predicted in microRNA.ORG database. The intersection of the predicted results from these four databases indicated that only ‐miR‐372‐3p (has‐miR‐372) was simultaneously predicted by all four databases (Figure 1E). These results demonstrated that miR‐372 played a regulatory role in NPC through modulation of PBK and the p53 signaling pathway.

**Table 2:** The NPC‐related known genes (Top 10)

Gene	Gene name	Score	PMIDs
TP53	tumor protein p53	0.224	78
CDKN2A	cyclin‐dependent kinase inhibitor 2A	0.206	24
CCND1	cyclin D1	0.206	23
PIK3CA	phosphatidylinositol‐4,5‐bisphosphate 3‐kinase catalytic subunit alpha	0.205	19
MMP2	matrix metallopeptidase 2	0.204	16
PTEN	phosphatase and tensin homolog	0.204	15
CLCN3	chloride voltage‐gated channel 3	0.202	8
ERCC1	ERCC excision repair 1, endonuclease non‐catalytic	0.202	8
KRAS	KRAS proto‐oncogene, GTPase	0.201	4
ERBB2	erb‐b2 receptor tyrosine kinase 2	0.201	3

Score of the reliability of the gene‐disease pair, based on the type and number of sources where is reported, and the number of pmids.

CCND1, cyclin D1; CDKN2A, cyclin‐dependent kinase inhibitor 2A; CLCN3, chloride voltage‐gated channel 3; ERBB2, erb‐b2 receptor tyrosine kinase 2; ERCC1, excision repair cross‐complementation group 1; KRAS, KRAS proto‐oncogene, GTPase; MMP2, matrix metallopeptidase 2; NPC, nasopharyngeal carcinoma; PIK3CA, phosphatidylinositol‐4,5‐bisphosphate 3‐kinase catalytic subunit alpha; PMIDs, total number of PMIDs supporting the association; PTEN, phosphatase and tensin homolog; TP53, tumor protein p53.

### 3.2 MiR‐372 targets PBK

**Figure 2:**
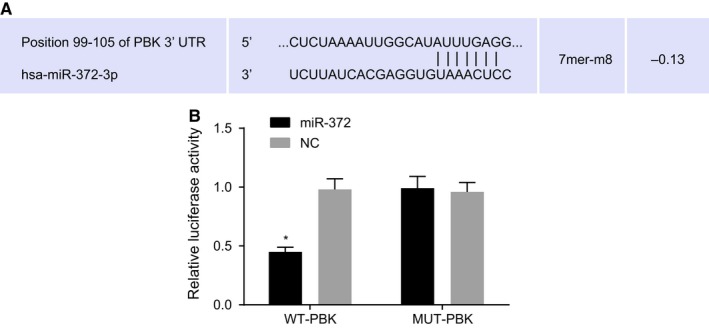
PBK is a target gene of miR‐372. A, predicted binding site between 3'‐UTR of PBK and miR‐372; B, luciferase activity of WT‐PBK and MUT‐PBK; ^*^ *P* < 0.05 vs the other three groups; miR‐372, microRNA‐372; PBK, PDZ‐binding kinase; UTR, untranslated region; WT, wild type; MUT, mutant; NC negative control

Bioinformatics online prediction and dual‐luciferase reporter gene assay were conducted in order to predict and verify the target relationship between miR‐372 and PBK (Figure 2). The results of bioinformatics online prediction showed that PBK was the potential target gene of miR‐372, and theoretically suggested that there were complementary sequences between 3′‐UTR of PBK and the seed zone of miR‐372. In addition, results of dual‐luciferase reporter gene assay revealed that co‐transfected WT‐PBK and miR‐372 showed obvious lower luciferase activity compared to other groups (*P* < 0.05), which indicated that miR‐372 significantly reduced the luciferase activity of WT‐PBK while exerted no obvious influence on the luciferase activity of MUT‐PBK. The aforementioned findings revealed that miR‐372 could specifically bind to PBK.

### 3.3 MiR‐372 and X‐ray radiation reduce NPC cell colony formation ability and proliferation activity

**Figure 3:**
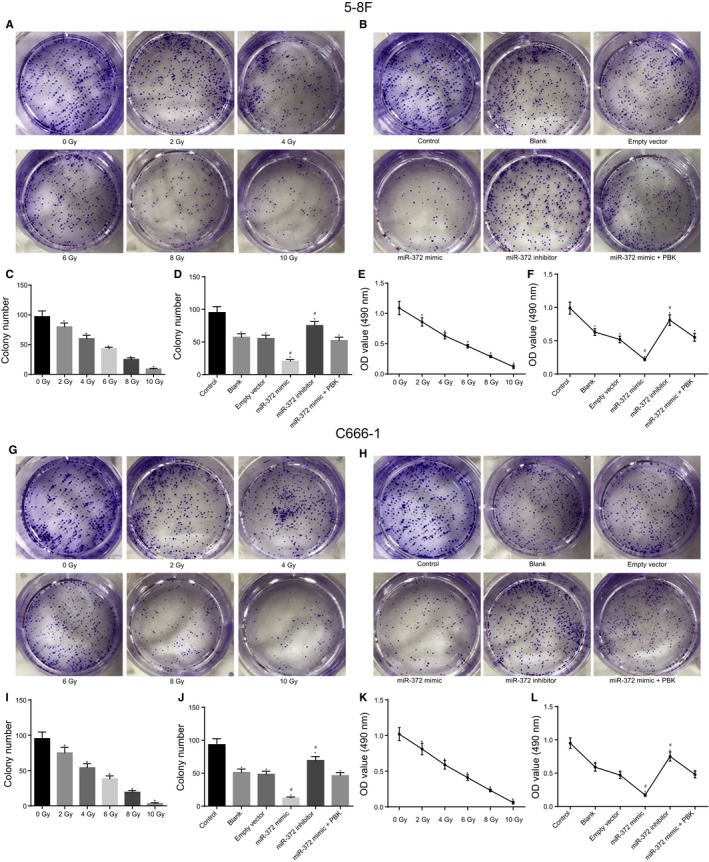
MiR‐372 and X‐ray radiation inhibit NPC cell clone formation and cell proliferation. A, C, G, and I, colony formation abilities of 5‐8F and C666‐1 cells after radiation of X‐ray with various dosage; ^*^, *P* < 0.05 vs 0 Gy; B, D, H, and J, colony formation abilities of 5‐8F and C666‐1 cells after radiation of X‐ray and alteration of miR‐372 and PBK expression, detected by clonogenic assay; ^*^ *P < *0.05 vs the control group; ^#^ *P* < 0.05 vs the blank and empty vector groups; E and K, cell viability of 5‐8F and C666‐1 cells after radiation of X‐ray examined by MTT; ^*^ *P* < 0.05 vs 0 Gy; F and L, cell viability of 5‐8F and C666‐1 cells after radiation of X‐ray and alteration of miR‐372 and PBK expression; ^*^ *P* < 0.05 vs the control group; ^#^ *P* < 0.05 vs the blank and empty vector groups. MTT, 3‐(4,5‐dimethylthiazol‐2‐yl)‐2,5‐diphenyltetrazolium bromide; miR‐372, microRNA‐372; NPC, nasopharyngeal carcinoma

Furthermore, the current study employed clonogenic assay in order to detect the effect of X‐ray radiation (with various dosages) and miR‐372 on the colony formation abilities of NPC cell lines 5‐8F and C666‐1. The colony formation abilities of 5‐8F and C666‐1 were found to be markedly reduced as X‐ray radiation was gradually increased in comparison with cells devoid of X‐ray radiation (Figure 3A,C,G,I). In addition, miR‐372 and its target gene PBK were observed to influence the colony formation abilities of 5‐8F and C666‐1 cells. Compared with the control group, the other groups showed significantly decreased colony formation abilities of 5‐8F and C666‐1 cells (*P < *0.05). No significant differences were observed in the colony formation abilities of 5‐8F and C666‐1 cells among the blank group and the empty vector group (*P* > 0.05). As compared to the blank and empty vector groups, the miR‐372 mimic group displayed reduced cell colony formation ability (*P* < 0.05), whereas the miR‐372 inhibitor group presented with increased cell colony formation ability (*P* < 0.05). Moreover, no significant differences in the colony formation abilities of 5‐8F and C666‐1 cells were observed among the blank, empty vector and miR‐372 mimic + PBK groups (*P* > 0.05) (Figure 3B,D,H,J). Therefore, the results revealed that miR‐372 and X‐ray radiation exposure decreased NPC cell colony formation ability, while restored PBK expression reversed the inhibitory effect of miR‐372 on colony formation.

Additionally, MTT assay was performed to measure the effect of X‐ray radiation on cell viability of 5‐8F and C666‐1 cells, and the results revealed that X‐ray radiation could inhibit the viability of 5‐8F and C666‐1 cells in a dosage‐dependent manner (Figure 3,E,K). The results showed that the other groups displayed significantly decreased cell viability in comparison with the control group (*P* < 0.05). No obvious cell viability changes were detected among the blank group and the empty vector group (*P* > 0.05). However, the miR‐372 mimic group exhibited reduced cell viability (*P* < 0.05), whereas the miR‐372 inhibitor group presented with increased cell viability (*P < *0.05) in comparison with the blank and empty vector groups. No significant cell viability differences were observed among the blank, empty vector and miR‐372 mimic +PBK groups (*P* > 0.05) (Figure 3,F,L). Therefore, the results revealed that X‐ray radiation contributed to inhibition of cell proliferation in a dosage‐dependent manner, and that miR‐372 also suppressed 5‐8F and C666‐1 cell proliferation, while restored PBK expression reversed the inhibitory effect of miR‐372 on cell proliferation.

### 3.4 Over‐expressed miR‐372 down‐regulates levels of PBK and Bcl‐2 while up‐regulates levels of p53, Caspase‐3, and Bax in NPC cells

**Figure 4:**
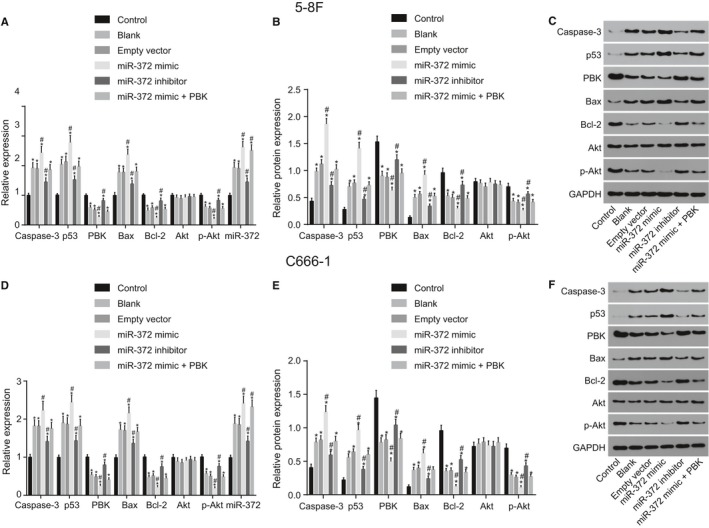
MiR‐372 over‐expression decreases protein levels of PBK and Bcl‐2 as well as the extent of Akt phosphorylation, while elevates protein levels of p53 and Bax. A and D, mRNA expression of p53 signaling pathway‐ and apoptosis‐related factors in 5‐8F and C666‐1 cells in each group examined by RT‐qPCR; B and E, the protein expression of p53 signaling pathway‐ and apoptosis‐related factors in 5‐8F and C666‐1 cells in each group examined by Western blot analysis; C and F, gray value of PBK, Bcl‐2, p53 and Bax, Akt, GAPDH as well as phosphorylated Akt in 5‐8F and C666‐1 cells in each group; ^*^ *P* < 0.05 vs the control group; ^#^ *P* < 0.05 vs the blank and empty vector groups; miR‐372, microRNA‐372; PBK, PDZ‐binding kinase; Bcl‐2, B‐cell lymphoma‐2; Bax, Bcl2 Associated X; Akt, protein kinase B; GAPDH, glyceraldehyde‐3‐phosphate dehydrogenase

RT‐qPCR and Western blot analysis were performed to detect the mRNA and protein levels of p53 signaling pathway‐ and apoptosis‐related factors (Figure 4). Results showed that compared with the control group, the other groups exhibited reduced mRNA and protein levels of PBK, Bcl‐2, and reduced Akt phosphorylation extent, however, mRNA and protein levels of p53, Bax, and Caspase‐3 were found to be increased (*P* < 0.05). In comparison with the blank group, the empty vector group displayed no obvious changes in mRNA and protein levels of the aforementioned factors (*P* > 0.05). In comparison with the blank group and empty vector groups, the miR‐372 mimic group exhibited reduced mRNA and protein levels of PBK, Bcl‐2, and Akt phosphorylation extent, while that of p53, Bax, and Caspase‐3 were increased in the miR‐372 mimic group, whereas opposite trends were found in the miR‐372 inhibitor group (all *P < *0.05). The protein levels of Akt remained unchanged among the blank, empty vector, miR‐372 mimic and miR‐372 inhibitor groups (*P* > 0.05). Additionally, no significant differences in the mRNA and protein levels of the aforementioned factors were detected among the blank, empty vector, and miR‐372 mimic + PBK groups (*P* > 0.05). Strikingly, the other groups were found to exhibit higher expression of miR‐372 in comparison with the control group (*P* < 0.05). In comparison with the blank group and empty vector groups, the miR‐372 expression was found to be remarkably increased in the miR‐372 mimic group, while decreased miR‐372 expression was observed in the miR‐372 inhibitor group (*P* < 0.05). Therefore, the results and findings revealed that over‐expression of miR‐372 down‐regulated levels of PBK, Bcl‐2, and extent of Akt phosphorylation while up‐regulated levels of p53, Bax, and Caspase‐3 in 5‐8F and C666‐1 cells. Furthermore, the elevation of PBK resulted in reversed effects on those gene expression.

### 3.5 X‐ray radiation and miR‐372 over‐expression promotes NPC cell apoptosis and induces cell arrest at G1 stage

**Figure 5:**
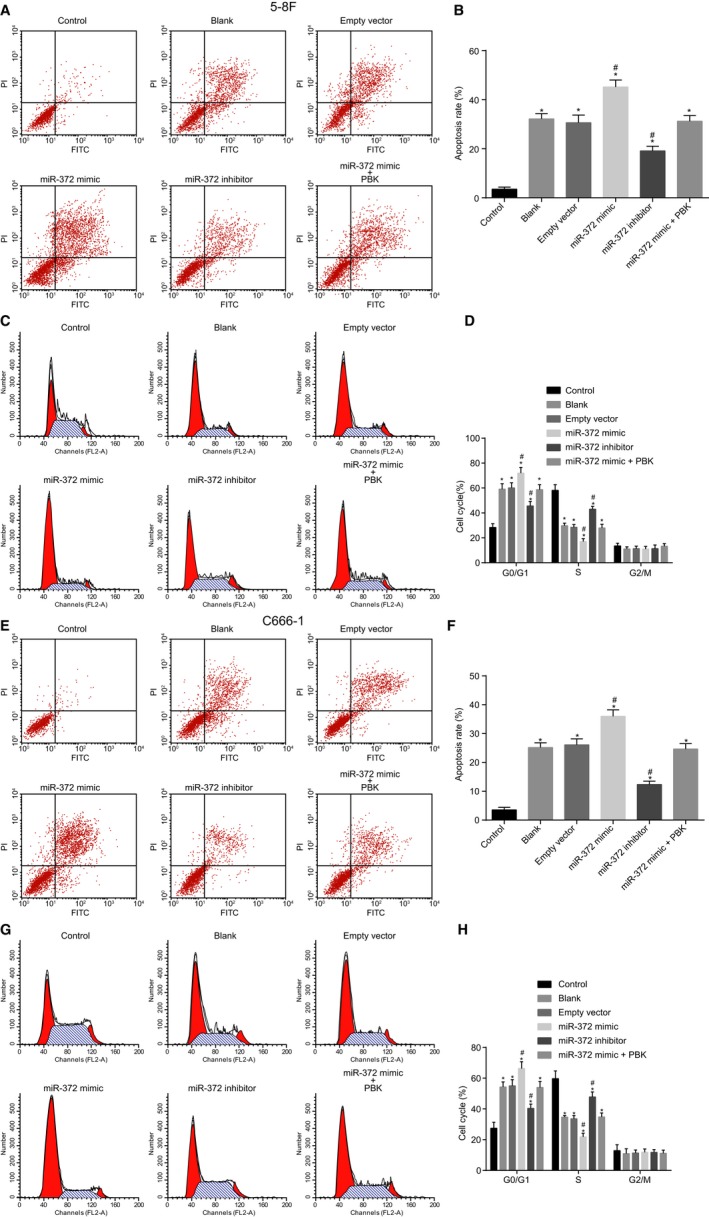
NPC cell apoptosis and cycle arrest are promoted by over‐expressed miR‐372 and X‐ray radiation. A and E, apoptosis of 5‐8F and C666‐1 cells detected by flow cytometry; B and F, apoptosis rate in 5‐8F and C666‐1 cells after radiation of X‐ray and alteration of miR‐372 and PBK expression; C and G, cell cycle distribution of 5‐8F and C666‐1 cells examined by PI staining; D and H, cell proportion at G1, S, and G2 stage in 5‐8F and C666‐1 cells after radiation of X‐ray and alteration of miR‐372 and PBK expression; ^*^ *P* < 0.05 vs the control group; ^#^ *P* < 0.05 vs the blank and empty vector groups; miR‐372, microRNA‐372; PI, propidium iodide; NPC, nasopharyngeal carcinoma

Annexin V‐FITC/PI staining was carried out in order to detect apoptosis of 5‐8F and C666‐1 cells. The results showed that the other groups displayed increased cell apoptosis in comparison with the control group (*P* < 0.05). In comparison with the blank group, the empty vector group showed no obvious changes in cell apoptosis, while the miR‐372 mimic group presented with increased cell apoptosis (*P* < 0.05) and the miR‐372 inhibitor group exhibited reduced cell apoptosis (*P* < 0.05). No significant differences in cell apoptosis were found among the blank, empty vector, and miR‐372 mimic +PBK groups (*P* > 0.05) (Figure 5A‐D). Therefore, the aforementioned results demonstrated that X‐ray radiation and miR‐372 over‐expression enhanced the apoptosis of 5‐8F and C666‐1 cells, while restored PBK expression reversed the promoting effect of miR‐372 on cell apoptosis.

Additionally, the current study employed PI staining combined with flow cytometry in order to measure the cell cycle of NPC cells with various treatments. The results showed that the proportions of 5‐8F cells at the G1 stage in the control, blank, empty vector, miR‐372 mimic, miR‐372 inhibitor, and miR‐372 mimic + PBK groups were (28.36 ± 2.98)%, (59.15 ± 4.32)%, (60.09 ± 4.14)%, (71.95 ± 4.63)%, (45.62 ± 3.62)%, and (58.69 ± 4.03)%, respectively; cell proportions at the S stage in those groups were (58.11 ± 4.63)%, (29.76 ± 2.06)%, (28.53 ± 2.17)%, (17.03 ± 2.34)%, (43.02 ± 2.27)%, and (27.99 ± 2.86)%, respectively; and cell proportions at the GA/M stage in those groups were (13.53 ± 2.15)%, (11.09 ± 1.68)%, (11.38 ± 2.01)%, (11.02 ± 2.12)%, (11.36 ± 2*.*95)%, and (13.32 ± 2.02)%, respectively. The proportions of C666‐1 cells at the G1 stage in the control, blank, empty vector, miR‐372 mimic, miR‐372 inhibitor, and miR‐372 mimic +PBK groups were (27.41 ± 3.96)%, (54.32 ± 3.26)%, (55.02 ± 4.02)%, (66.32 ± 4.35)%, (40.36 ± 2.82)%, and (53.98 ± 3.94)%, respectively; cell proportions at the S stage in those groups were (59.72 ± 4.96)%, (34.69 ± 1.05)%, (33.61 ± 2.11)%, (21.84 ± 2.39)%, (47.85 ± 3.15)%, and (34.86 ± 2.48)%; and cell proportions at the G2/M stage in those groups were (12.87 ± 3.86)%, (10.99 ± 3.12)%, (11.37 ± 1.94)%, (11.84 ± 2.12)%, (11.79 ± 1.79)%, and (11.16 ± 2.08)%, respectively. In contrast to the control group, the blank, empty vector, miR‐372 mimic, miR‐372 inhibitor, and miR‐372 mimic + PBK groups exhibited increased cells at G1 stage and reduced cells at S stage (*P* < 0.05). No significant differences were noted among the blank group and the empty vector group (*P* > 0.05). Compared with the blank group and the empty vector group, the miR‐372 mimic group exhibited increased cells at G1 stage and decreased cells at S stage while the miR‐372 inhibitor group exhibited opposite trends (all *P* < 0.05). No significant differences regarding the cell cycle were found among the blank, empty vector, and miR‐372 mimic + PBK groups (*P* > 0.05) (Figure 5E‐H). These findings demonstrated that over‐expressed miR‐372 induced NPC cell cycle arrest, while restored PBK expression reversed the promoting effect of miR‐372 on cell cycle arrest.

### 3.6 MiR‐372 up‐regulation and X‐ray reduce NPC cell migration and invasion

**Figure 6:**
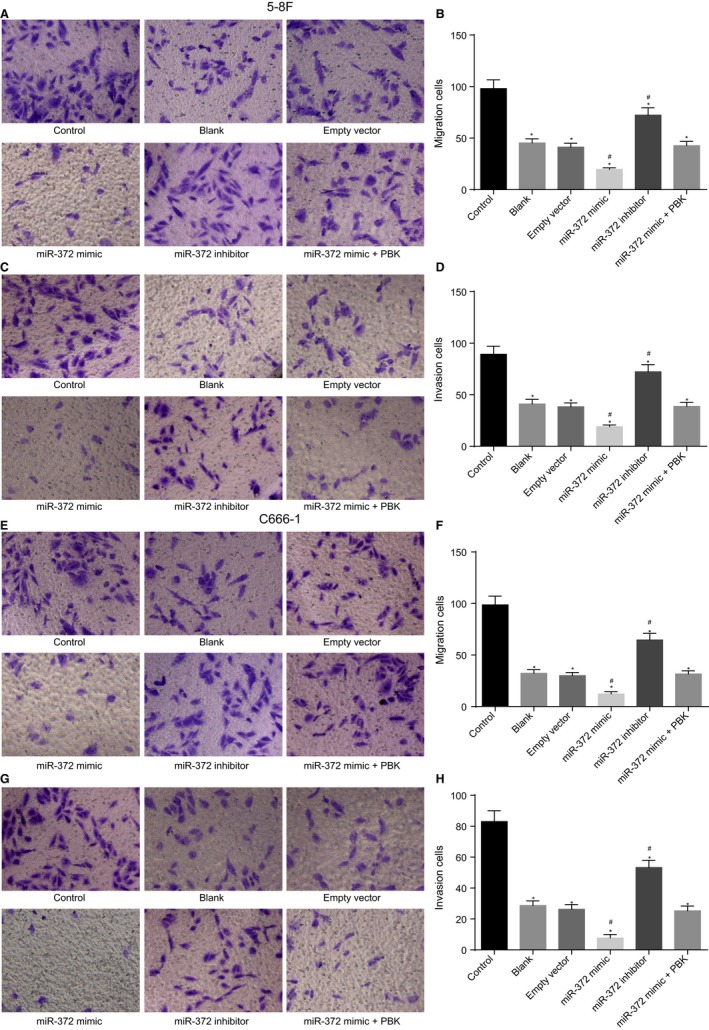
MiR‐372 elevation and X‐ray radiation inhibit NPC cell migration and invasion. A and E, the cell migration examined by Transwell assay; B and F, the number of migration cells after different treatments; C and G, invasion of 5‐8F and C666‐1 cells in each group examined by Transwell assay; D and H, the number of invasion cells after different treatments. ^*^ *P* < 0.05 vs the control group; ^#^ *P* < 0.05 vs the blank and empty vector groups; miR‐372, microRNA‐372; NPC, nasopharyngeal carcinoma

Transwell assay was performed in order to determine cell migration and invasion of 5‐8F and C666‐1 cells. The results (Figure 6) showed that in contrast to the control group, the blank, empty vector, miR‐372 mimic, miR‐372 inhibitor, and miR‐372 mimic + PBK groups presented with reduced cell migration and invasion abilities (*P* < 0.05). No obvious cell migration and invasion differences were found among the blank group and the empty vector group (*P* > 0.05). The cell migration and invasion abilities were decreased in the miR‐372 mimic group (*P* < 0.05), while opposite trends were observed in the miR‐372 inhibitor group (*P* < 0.05) compared with the blank and empty groups. No significant cell invasion differences were observed among the blank, empty vector, and miR‐372 mimic + PBK groups (*P* > 0.05). Therefore, these findings manifested that X‐ray radiation and elevated miR‐372 contributed to suppression of NPC cell migration and invasion.

### 3.7 Over‐expressed miR‐372 and radiotherapy suppress tumor growth in NPC

**Figure 7:**
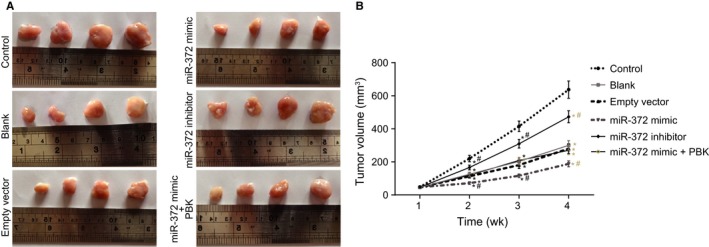
Tumor growth in NPC is inhibited by up‐regulated miR‐372 and radiotherapy. A, formed tumors in rats with injection of 5‐8F cells after different treatments; B, tumor volume in rats with injection of 5‐8F cells after different treatments; ^*^ *P* < 0.05 vs the control group; ^#^ *P* < 0.05 vs the blank and empty vector groups; miR‐372, microRNA‐372; NPC, nasopharyngeal carcinoma

The in vitro experimentation revealed that miR‐372 inhibition could promote cell proliferation and migration and reduce radiosensitivity. The current study adopted tumor xenografts in nude mice to evaluate the effect of miR‐372 on enhancing radiosensitivity. Results revealed that the volume of tumor in other groups was reduced in comparison with the control group (all *P* < 0.05). There were no significant differences in tumor volume among the blank group and the empty vector group (*P* > 0.05). In comparison with the blank group and the empty vector group, the volume of tumor in the miR‐372 mimic was obviously decreased (*P* < 0.05), while that in the miR‐372 inhibitor group was found to be increased (*P < *0.05). In comparison with the blank group, no obvious change was found in the empty vector group (*P* > 0.05). No significant differences in tumor volume were found among the blank, empty vector, and miR‐372 mimic + PBK groups (*P* > 0.05) (Figure 7). The above findings indicated that elevated miR‐372 and radiotherapy inhibited tumor growth in NPC, while the restored PBK expression reversed the anti‐tumor effect of miR‐372.

## 4 DISCUSSION

Nasopharyngeal carcinoma is regarded as a common tumor in Africa and Southeastern Asia, which is highly associated with genetic factor, dietary impact, and viral infection.^22^ Currently, chemotherapy remains to be the main therapeutic treatment of choice for NPC patients.^3^ However, numerous factors including relapse, distant metastasis, and resistance to radiation reduce the efficacy of radiotherapy on NPC treatment, thus a new therapeutic regimen aiming to enhance the radiosensitivity of NPC could improve the overall survival rate and quality of life of NPC patients.^4^ Interestingly, a recent study revealed that miRs play a regulatory role in tumor radiosensitivity from several perspectives, such as cell apoptosis and the DNA damage repair modulation.^5^ Moreover, miRs have been reported to be related to NPC progression, and its abnormal expression is correlated with the regulation of tumor progression, such as tumor formation, metastasis, and proliferation indicating toward unfounded roles of miRs in NPC.^23^, ^24^ Collectively, findings from the current study revealed that over‐expressed miR‐372 enhances radiosensitivity while inhibits invasion and metastasis by down‐regulating PBK and activating the p53 signaling pathway in NPC.

Initially, NPC‐related DEGs analyzed by microarray gene expression profiling and miRs regulating PBK predicted online demonstrated that miR‐372 played a regulatory role in NPC through modulating PBK. Then, based on the target prediction program and the dual‐luciferase activity determination, PBK was found to be a target gene of miR‐372. A recent study revealed that the expression of miR‐372 was significantly decreased in NPC TW01 cells.^25^ In addition, up‐regulated levels of PBK have been previously found in NPC, and it’s up‐regulation was reported be to associated with poor survival rates, worse tumor development, and advanced T‐stage in patients with NPC.^6^ Similarly, miR‐216b has been proved to promote chemosensitivity and inhibit cell proliferation by restraining PBK in colorectal cancer (CRC), which provided useful insights for the treatment of CRC.^7^ Meanwhile, miR‐770‐5p, a novel miR induced by radiation, has been previously demonstrated to enhance tumor radiosensitivity by direct modulating PBK.^26^ The above findings are in line with our results reflecting that miR‐372 targets PBK, and miR‐372 negatively regulates PBK.

In addition, we found that over‐expression of miR‐372 leads to decreased mRNA and protein levels of Bcl‐2 and the extent of Akt phosphorylation while increased that of p53, Caspase‐3, and Bax, indicating that miR‐372 elevation could promote apoptosis and activate the p53 signaling pathway. Bax, a protein encoded by nuclear in higher eukaryotes, possesses the ability to regulate apoptosis‐induced cell death.^27^ Moreover, Bax is regarded as a novel therapeutic biomarker for NPC treatment, and a previous study found that over‐expression of Bax enhances radiosensitivity in NPC patients at advanced stages.^28^ Interestingly, a recent study demonstrated that Bax can be up‐regulated by miR‐372.^29^ Bcl‐2, the founding member of the Bcl‐2 family of regulator proteins, is involved in the regulation of apoptosis, and its elevated expression has been found in multiple human cancers.^30^ Remarkably, a previous study revealed that miR‐372 can cause down‐regulation of Bcl‐2.^31^ In addition, activated Akt is related to NPC metastasis, and its knockdown evidently enhances cell apoptosis of SUNE‐1 cells in NPC.^32^ MiR‐330 over‐expression enhances cell apoptosis *via* inhibiting E2F1‐mediated Akt phosphorylation in prostate cancer.^33^ Another study reported that NPC cell apoptosis is induced by activated p53‐p21 signaling pathway through down‐regulated long noncoding RNA highly increased in liver cancer.^19^ Thereby, it can be inferred that miR‐372 improved cell apoptosis in NPC by activating the p53 signaling pathway. Moreover, we found that restoration of the expression of PBK up‐regulated the expression of Bcl‐2 but reduced that of p53, Caspase‐3, and Bax, suggesting that PBK inhibited apoptosis of NPC cells, and reversed the miR‐372‐induced activation of the p53 signaling pathway. Previously, it has been revealed that PBK attenuates DNA damage checkpoint through suppression of p53, thus augmenting tumor cell growth.^34^ Also, increased PBK promoted the progression of tumor cells by inhibiting p53.^35^ Hence, it is reasonable to conclude that miR‐372 targets PBK to down‐regulate its expression, thus preventing the development of NPC.

Furthermore, our findings evidence that up‐regulation of miR‐372 and radiotherapy inhibits NPC cell colony formation, proliferation, tumor growth, migration, and invasion while promoting cell apoptosis. Similarly, a recent study found that miR‐372 is associated with a decrease in cell cycle entry and increased cell apoptosis in NPC TW01 cells, revealing that miR‐372 could serve as a tumor suppressor in NPC cells.^25^ In addition, tumor cell invasion and proliferation were found to be restrained by miR‐372 *via* IGF2BP1 in renal cell carcinoma.^9^ Additionally, the current study further explored the effect of miR‐372 on radiosensitivity, which suggested that up‐regulation of miR‐372 promoted radiosensitivity. In line with our study, another study demonstrated that over‐expression of miR‐24 promotes radiosensitivity via binding to specificity protein 1 in NPC.^36^ Also, miR‐206 has been demonstrated to increase NPC radiosensitivity via regulating IGF1.^37^ Moreover, over‐expression of PBK was revealed to contribute to increased colony formation, migration and invasion in NPC cells and tumor growth, while decreasing radiosensitivity in the current study. Up‐regulation of PBK could improve the growth of lung adenocarcinoma cells,^38^ and promoted the progression of esophageal squamous cell carcinoma.^39^ Similarly, miR‐770‐5p was shown to enhance radiosensitivity by suppressing PBK.^26^ Thus, the above‐mentioned results suggest that over‐expression of miR‐372 enhances radiosensitivity while inhibiting invasion and metastasis by negative regulation of PBK and activation of the p53 signaling pathway in NPC.

**Figure 8:**
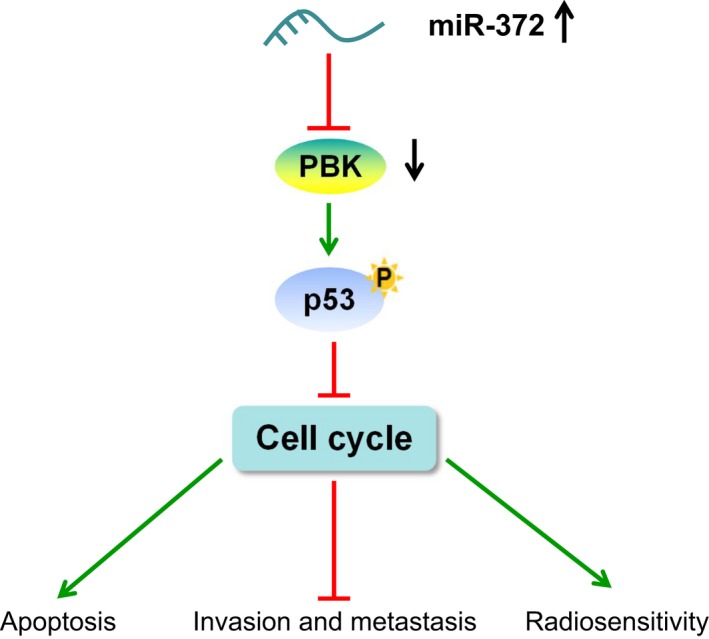
The molecular mechanism involved in miR‐372 and PBK regulating NPC through p53 signaling pathway. Over‐expressed miR‐372 promotes radiosensitivity while inhibits invasion and metastasis in NPC. MiR‐372 over‐expression could inhibit PBK expression and then activate the p53 signaling pathway, thus enhancing radiosensitivity while suppressing invasion and metastasis in NPC; miR‐372, microRNA‐372; NPC, nasopharyngeal carcinoma; PBK, PDZ‐binding kinase

In conclusion, the current study revealed that miR‐372 contributes to enhanced radiosensitivity while represses invasion and metastasis in NPC. The enhanced miR‐372 expression inhibits the expression of PBK, and further activates the p53 signaling pathway, thus enhancing radiosensitivity while suppressing invasion and metastasis in NPC (Figure 8). These findings may serve as novel ideas for future NPC treatments. However, due to limited time, space and study subjects, this study was not so comprehensive and warranted further exploration. It is expected that further studies involving the mechanism of miR‐372, PBK, and the p53 signaling pathway on other cancer types will be conducted in the future in order to achieve better treatment regimens for NPC.

## CONFLICT OF INTEREST

None declared.
