# Corrigendum

**DOI:** 10.1002/cam4.4489

**Published:** 2021-12-14

In the article by Zhe et al.,^1^ entitled “MicroRNA‐372 enhances radiosensitivity while inhibiting cell invasion and metastasis in nasopharyngeal carcinoma through activating the PBK‐dependent p53 signaling pathway,” the author wants to correct the misapplied figure 5. Please find the corrected Figure 5 below.

**FIGURE 5:**
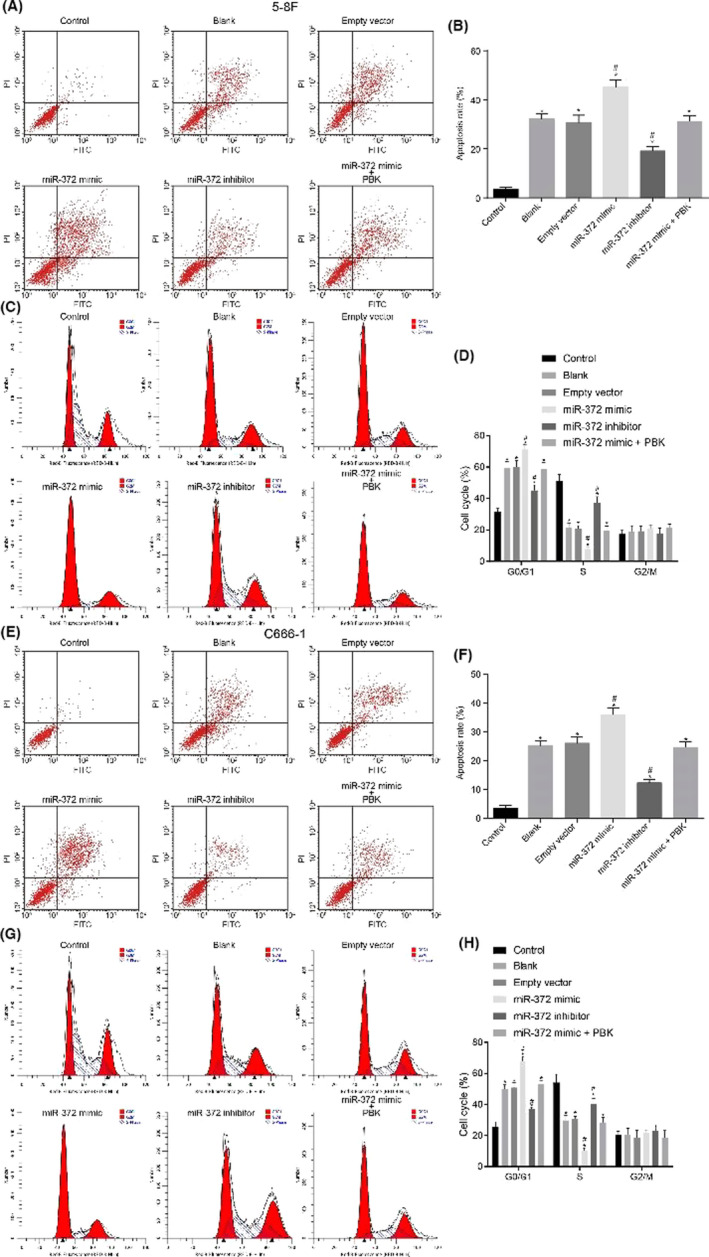
NPC cell apoptosis and cycle arrest are promoted by over‐expressed miR‐372 and X‐ray radiation. A and E, apoptosis of 5‐8F and C666‐1 cells detected by flow cytometry; B and F, apoptosis rate in 5‐8F and C666‐1 cells after radiation of X‐ray and alteration of miR‐372 and PBK expression; C and G, cell cycle distribution of 5‐8F and C666‐1 cells examined by PI staining; D and H, cell proportion at G1, S, and G2 stage in 5‐8F and C666‐1 cells after radiation of X‐ray and alteration of miR‐372 and PBK expression; *p < 0.05 versus the control group; #*p* < 0.05 versus the blank and empty vector groups; miR‐372, microRNA‐372; PI, propidium iodide; NPC, nasopharyngeal carcinoma
